# Recurrence of Post-dural Puncture Headache After a Successful Blood Patch

**DOI:** 10.7759/cureus.48497

**Published:** 2023-11-08

**Authors:** João Barbosa, Maria Valentim, Mariana Almeida, Sara Carneiro, Lúcia Vasconcelos

**Affiliations:** 1 Anesthesiology Department, Hospital de Braga, Braga, PRT

**Keywords:** unintended dural puncture, cerebrospinal fluid hypotension, subdural hematomas, subdural hygromas, post-dural puncture headache, epidural blood patch

## Abstract

The obstetric population is at a higher risk of experiencing post-dural puncture headache (PDPH), which is a frequent complication that can occur following spinal anesthesia or unintended dural puncture during epidural catheter placement. If conservative treatment fails to resolve symptoms, the epidural blood patch (EBP) is the definitive treatment for PDPH.

We present the case of a 35-year-old nulliparous woman who developed PDPH and underwent treatment with an EBP. There was immediate resolution of symptoms and she was discharged home. However, three days later, the symptoms recurred, and subdural hygromas were found on a cerebral CT scan.

This case report emphasizes the importance of a multidisciplinary approach involving anesthesiology, obstetrics, and neurology in managing PDPH and the associated complications in postpartum patients.

## Introduction

Post-dural puncture headache (PDPH) is one of the most frequent complications following spinal anesthesia or unintentional dural puncture (UDP) during epidural catheter placement in obstetric patients [1]. UDP occurs in 1.5% of the obstetric population [1], and 81-88% of those patients report PDPH [2]. This high incidence of symptoms corroborates the increased risk for PDPH in females, with additional risk during pregnancy. The epidural blood patch (EPB) is considered the definitive treatment for PDPH. It usually provides immediate relief and has a success rate of around 90%. Additionally, 90% of those who do not improve with the first EPB improve after the second [3]. The recurrence of PDPH after a first successful EBP with complete resolution of symptoms is rare but has been described before [4].

This case presents a spontaneous recurrence of PDPH one week after a successful EBP, which is an uncommon condition.

## Case presentation

A healthy 35-year-old nulliparous woman, American Society of Anesthesiologists class 2, received an epidural catheter for labor analgesia. There was an accidental puncture of the dura mater with an 18 G Tuohy needle in the intervertebral space L3-L4. Conservative treatment with non-steroidal anti-inflammatory drugs (NSAIDs), paracetamol, hydration, and caffeine was immediately started. On postpartum day one, she developed a severe and incapacitating headache consistent with PDPH and underwent treatment with an EPB in the intervertebral space L4-L5. Complete resolution was observed minutes after the procedure with the administration of 17 mL of autologous blood until the patient reported a slight sensation of lumbar pressure. She was discharged in an asymptomatic state on postpartum day two while continuing conservative treatment. On postpartum day five, she presented to the emergency department with a recurrence of severe headache. She continued taking paracetamol and NSAIDs, increased hydration, and caffeine, but there was no improvement.

On neurological examination, no deficits were observed. The patient denied any visual changes, photophobia, nausea, or vomiting. The patient agreed to be readmitted for further conservative treatment until the following day. In addition to the ongoing treatment, a sphenopalatine ganglion block was performed with mild improvement. On postpartum day six, Neurology was consulted and recommended a cerebral CT scan to rule out complications related to intracranial hypotension due to cerebrospinal fluid leak. The patient was unable to tolerate an upright position and continued to experience debilitating symptoms. In addition to the cerebral CT scan, a repeat complete blood count and coagulation study were requested to assess the neuroaxis if necessary.

**Figure 1:**
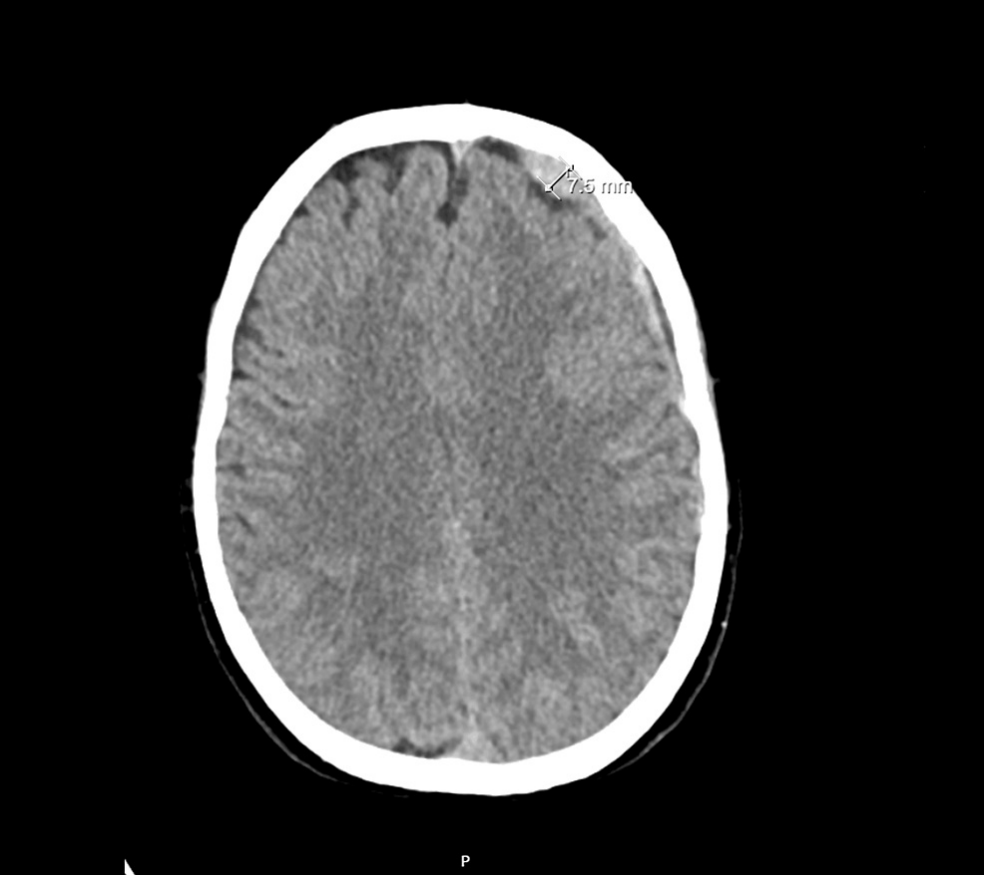
The patient’s cerebral CT scan revealed the presence of hygromas in the context of intracranial hypotension.

The cerebral CT scan revealed the presence of hygromas in the context of intracranial hypotension with larger dimensions on the left side, reaching a maximum thickness of approximately 7 mm (Figure 1).

Additionally, there was a predominantly left-sided hematoma component under the tentorium cerebelli. These findings were associated with a reduction in the size of the ventricular dimensions, which is consistent with the clinical information of PDPH.

Neurology advised that, given the severe symptoms and imaging findings, the patient would benefit from a second EBP to manage her intracranial hypotension. The procedure was explained again to the patient, and a new informed consent was obtained. The technique was performed again without complications in the intervertebral space L4-L5, with the administration of 18 mL of autologous blood, until the patient reported a slight sensation of lumbar pressure.

On postpartum day seven, one day after the second EBP, the patient was asymptomatic and could tolerate an upright position. It was suggested to continue the oral analgesia regimen for the next three days, and the patient was discharged from the hospital.

A follow-up phone call was made six days after the second EBP procedure, and the patient reported being asymptomatic and fully resumed her daily activities since the discharge.

## Discussion

The risk of PDPH following accidental dural puncture is approximately 80% [2], and the majority of cases resolve with conservative treatment [5]. However, in some cases, conservative treatment is insufficient to alleviate severe symptoms, and the definitive treatment for PDPH is an EBP [6]. The literature describes a success rate of up to 86% after the first procedure [2], and a second EBP is usually effective in cases where the initial procedure was unsuccessful.

However, the situation described is uncommon as there was a recurrence of symptoms after an initial successful EBP with complete resolution of symptoms for the patient.

The development of subdural hematomas in the context of intracranial hypotension due to a cerebrospinal fluid leak can be attributed to a decrease in cerebrospinal fluid volume and subsequent stretching and tearing of the bridging veins, which are concerning findings related to the severity of the symptoms [7].

The incidence of subdural hygromas following dural puncture remains unknown due to a lack of imaging investigation. In this case, the cerebral CT scan was done after symptom recurrence to rule out complications, but normally, as PDPH is a clinical diagnosis, most cases do not undergo any imaging investigation.

After the improvement in the second procedure, it was discussed with Neurology whether a follow-up CT scan was necessary. It was not considered necessary because there was a defined etiology for the imaging findings that was resolved by the procedure with symptomatic resolution.

It is important to emphasize the role of Obstetrics in the diagnosis and early recognition of these complications and their referral to Anesthesiology, as initially, these obstetric patients are triaged for obstetric emergency care. Interdisciplinary patient care in the obstetric population should be encouraged and valued to achieve the best outcome for the patient who needs to have a functional day-to-day life without limitations due to these symptoms to care for her newborn.

## Conclusions

The recurrence of symptoms after an effective EBP in a postpartum patient is rare, with an undefined prevalence in the literature. This case has prompted us to consider the need for a brain CT scan to rule out potential complications before considering a second approach and the implementation of follow-up consultations for these patients.

This case report underscores the importance of a multidisciplinary approach involving Anesthesiology, Obstetrics, and Neurology in managing PDPH and the associated complications in postpartum patients.
